# The Nephroprotective Effect of *Zizyphus lotus* L. (Desf.) Fruits in a Gentamicin-Induced Acute Kidney Injury Model in Rats: A Biochemical and Histopathological Investigation

**DOI:** 10.3390/molecules26164806

**Published:** 2021-08-08

**Authors:** Noureddine Bencheikh, Mohamed Bouhrim, Loubna Kharchoufa, Omkulthom Mohamed Al Kamaly, Hamza Mechchate, Imane Es-safi, Ahmed Dahmani, Sabir Ouahhoud, Soufiane El Assri, Bruno Eto, Mohamed Bnouham, Mohammed Choukri, Mostafa Elachouri

**Affiliations:** 1 Laboratory of Bioresources, Biotechnology, Ethnopharmacology and Health, Faculty of Sciences, Mohammed First University, B.P. 717, Oujda 60040, Morocco; bencheikh_noureddine1718@ump.ac.ma (N.B.); mohamed.bouhrim@gmail.com (M.B.); l.kharchoufa@ump.ac.ma (L.K.); dahmani.ahmed@ump.ac.ma (A.D.); ouahhoud.sabir@ump.ac.ma (S.O.); bnouham.mohamed@ump.ac.ma (M.B.); elachourimostafa@gmail.com (M.E.); 2 Department of Pharmaceutical Sciences, College of Pharmacy, Princess Nourah Bint Abdulrahman University, Riyadh 11564, Saudi Arabia; 3 Laboratory of Biotechnology, Environment, Agrifood and Health, Faculty of Sciences, University of Sidi Mohamed Ben Abdellah, Fez 30050, Morocco; imane.essafi1@usmba.ac.ma; 4 Faculty of Medicine and Pharmacy, Mohammed First University, B.P. 724, Oujda 60000, Morocco; elassri.soufiane@ump.ac.ma (S.E.A.); choukri.mohammed@ump.ac.ma (M.C.); 5 Laboratories-TBC, Faculty of Pharmaceutical and Biological Sciences, B.P. 83, 59000 Lille, France; titisfrance@gmail.com; 6 Biochemistry Laboratory, Central Laboratory Service—CHU, Mohammed VI University Hospital, B.P. 4806, Oujda 60049, Morocco

**Keywords:** *Zizyphus lotus* L., gentamicin, nephrotoxicity, protective effects, natural compounds, medicinal plants

## Abstract

*Zizyphus lotus* L. (Desf.) (*Z. lotus*) is a medicinal plant largely distributed all over the Mediterranean basin and is traditionally used by Moroccan people to treat many illnesses, including kidney failure. The nephrotoxicity of gentamicin (GM) has been well documented in humans and animals, although the preventive strategies against it remain to be studied. In this investigation, we explore whether the extract of *Zizyphus lotus* L. (Desf.) Fruit (ZLF) exhibits a protective effect against renal damage produced by GM. Indeed, twenty-four *Wistar* rats were separated into four equal groups of six each (♂/♀ = 1). The control group was treated orally with distilled water (10 mL/kg); the GM treated group received distilled water (10 mL/kg) and an intraperitoneal injection of GM (80 mg/kg) 3 h after; and the treated groups received ZLF extract orally at the doses 200 or 400 mg/kg and injected intraperitoneally with the GM. All treatments were given daily for 14 days. At the end of the experiment, the biochemical parameters and the histological observation related the kidney function was explored. ZLF treatment has significantly attenuated the nephrotoxicity induced by the GM. This effect was indicated by its capacity to decrease significantly the serum creatinine, uric acid, urea, alkaline phosphatase, gamma-glutamyl-transpeptidase, albumin, calcium, sodium amounts, water intake, urinary volume, and relative kidney weight. In addition, this effect was also shown by the increase in the creatinine clearance, urinary creatinine, uric acid, and urea levels, weight gain, compared to the rats treated only with the GM. The hemostasis of oxidants/antioxidants has been significantly improved with the treatment of ZLF extract, which was shown by a significant reduction in malondialdehydes levels. Histopathological analysis of renal tissue was correlated with biochemical observation. Chemical analysis by HPLC-DAD showed that the aqueous extract of ZLF is rich in phenolic compounds such as 3-hydroxycinnamic acid, catechin, ferulic acid, gallic acid, hydroxytyrosol, naringenin, p- coumaric Acid, quercetin, rutin, and vanillic acid. In conclusion, ZLF extract improved the nephrotoxicity induced by GM, through the improvement of the biochemical and histological parameters and thus validates its ethnomedicinal use.

## 1. Introduction

Kidney function is essential for maintaining the overall hemostasis of our body. This vital organ participates in the equilibrium of several important physiological functions such as detoxification, regulation of the acid-base and hydro-mineral balance, the regulation and the synthesis of some hormones, in particular, the erythropoietin necessary for the hematite synthesis, and blood pressure regulation [1]. By these facts related to these multiple functions, especially the detoxification property, the kidneys remain the most exposed organ in our body to different xenobiotics. Moreover, in clinical practice, several drugs were proved to be nephrotoxic [2]. As a matter of fact, in hospitalized patients, approximately 20% of the acute renal insufficiencies are due to the use of nephrotoxic drugs [3]. Many antibiotics, including tetracyclines, sulfonamides, beta-lactams, fluoroquinolones, vancomycin, daptomycin, and aminoglycosides, can adversely affect kidney function [4,5,6]. Aminoglycoside antibiotics (gentamicin (GM)) are often used to manage diseases of the urinary tract and abdomen [7]. However, it was documented that up to 30% of patients treated for more than 7 days with GM had some symptoms of nephrotoxicity (induced proximal tubular lesions) [8,9,10]. GM is the most nephrotoxic antibiotic in the aminoglycoside class, and its toxicity is externalized at the lowest dose [11,12,13]. The GM is associated with the production of reactive oxygen species in the form of superoxide anion (O^2−^), hydrogen peroxide (H_2_O_2_), and hydroxyl radical (OH•) of renal cortical mitochondria, which are accompanied by an increase in lipid peroxidation [11]. In the context of this concern, natural resources such as medicinal plants provide a reservoir of natural antioxidants can be used as a treatment to attenuate the nephrotoxicity produced by the drugs that stimulating oxidative stress. 

For this reason, *Zizyphus lotus* L. (Desf.) was chosen for its medicinal properties. It is a frequently used plant by the Moroccan people to treat several ailments, including nephrotoxicity [14,15,16]. This plant is commonly called “*Sadra*” in traditional Moroccan medicine and belongs to the *Rhamnaceae* family, wide-stretching in arid and semi-arid regions [17]. Various parts of this plant are traditionally used to manage a variety of health issues such as urinary tract infections, liver disorders, digestive problems, insomnia, diabetes, and skin infections [18]. Several pharmacological effects of this species have been confirmed such as Antiulcerogenic [19], anti-inflammatory, analgesic [20], antispasmodic [21], antidiabetic [22], gastro-protective [23], litholytic effects [24], and hepatoprotective [25]. Moreover, this plant has shown an important antioxidant activity, and the chemical analysis of this plant has shown its richness of antioxidant molecules [26]. So, this plant can be used as a natural product to trap free radicals produced by GM, which causes nephrotoxicity. However, there is no pharmacological investigation related to the potential nephroprotective of ZLF. In this respect, we undertook this study intending to evaluate the nephroprotective potential of the aqueous ZLF extract against the nephrotoxicity produced by the GM treatment in *Wistar* rats.

## 2. Results

### 2.1. Phytochemical Analysis of ZLF’s Aqueous Extract

**Figure 1:**
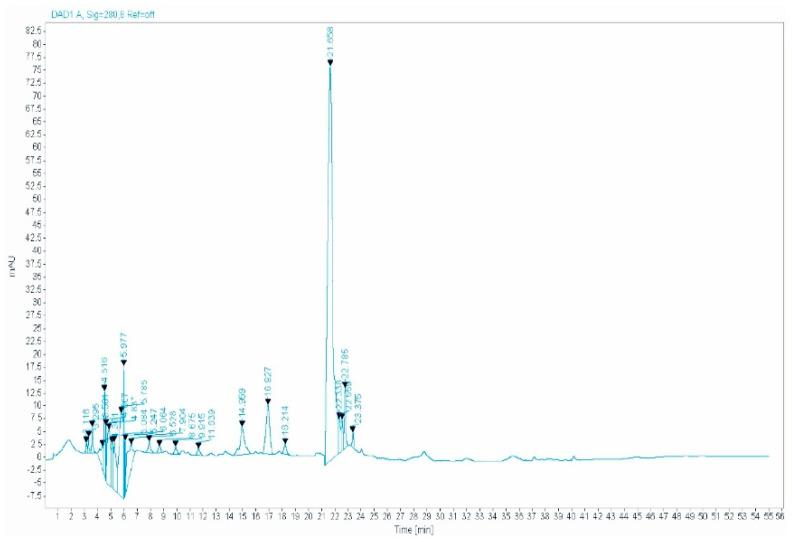
The HPLC chromatogram of ZLF aqueous extract.

Ten phenolic compounds were found in the aqueous extract of ZLF using the HPLC-DAD method (Table 1 and Figure 1). The quantities of polyphenols in the aqueous ZLF extract ranged from 2.21 to 137 μg/mL. The amount of ferulic acid in the ZLF was comparatively high, up to 137 μg/mL, followed by quercetin with 8.55 μg/mL. However, the minimal concentration was reported for hydroxytyrosol 2.21 μg/mL (Table 1).

### 2.2. Evaluation of the Nephroprotective Activity of ZLF Aqueous Extract

In this study, male and female rats were used to assess the nephroprotective effect of ZLF aqueous extract. After making a statistical comparison between the male and female sex of the same group in terms of the results of the biochemical parameters concerned in this study, it appears that no difference was observed between the two sexes. In other ways, sex does not influence the parameters that will be treated below.

#### 2.2.1. Effect of ZLF’s on Urine Volume and Water Intake

**Figure 2:**
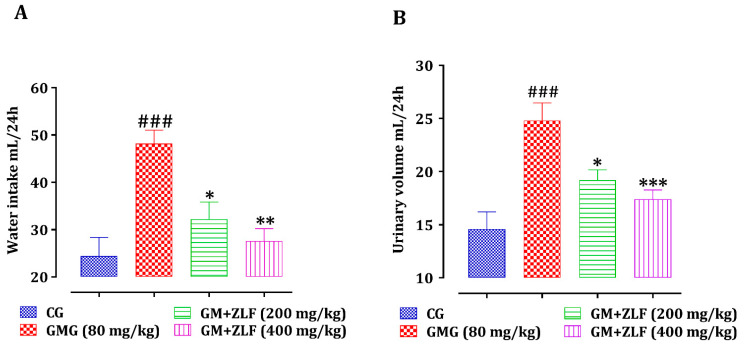
Effect of ZLF’s on intake of water (**A**) and the volume of urine (**B**) in GM-poisoned rats. The data are presented as mean ± SEM, (*n* = 6). ^###^ *p* ≤ 0.001 against the CG. * *p* < 0.05, ** *p* < 0.01, *** *p* < 0.001 against GMG. GM: gentamicin; CG: Control Group; GMG: GM treated Group.

ZLF were tested for their impact on the consumption of water and the volume of urine excreted in rats subjected to GM (Figure 2). In contrast to rats in the CG (Control Group), rats injected with the GM drug had a substantial improvement (*p* < 0.001) in urine production and urinary volume. Nonetheless, the treatment with ZLF’s at both studied doses, in conjunction with an intraperitoneal injection of GM (80 mg/kg; b.w), showed a significant reduction in water intake and urinary volume.

#### 2.2.2. Effect of ZLF’s on Weight Gain and Relative Kidney Weight

The effects of the ZLF on body weight gain and relative kidney weight are described in Table 2. By referring to CG, the regular intraperitoneal injection of GM (80 mg/kg; b.w) to the rats resulted in a substantial (*p* < 0.001) reduction in weight gain and a significant (*p* < 0.001) increase in total kidney weight. Nonetheless, each day’s pretreatment of the rats by ZLF’s at both studied doses, 3 h before the injection of GM, prevented the variation of these parameters, non-significant and significant (*p* < 0.01) increase in weight gain, respectively, compared to rats exposed only to GM. The relative kidney weights for the groups poisoned by GM (80 mg/kg; b.w) and treated with ZLF’s at both doses were significantly reduced (*p* < 0.01) and (*p* < 0.001), respectively.

#### 2.2.3. The Impact of ZLFs on Serum Creatinine, Uric Acid, and Urea Levels

**Figure 3:**
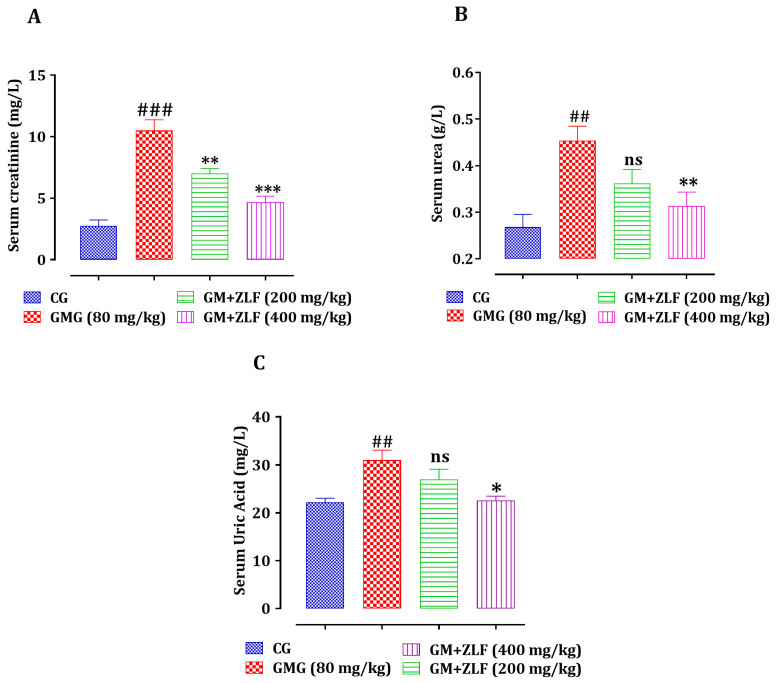
Effect of ZLF’s on serum creatinine (**A**), urea (**B**), and uric acid (**C**) in GM-poisoned rats. The data are presented as mean ± SEM, (*n* = 6). ^###^ *p* ≤ 0.001, ^##^ *p* ≤ 0.01 versus CG. * *p* < 0.05, ** *p* < 0.01, *** *p* < 0.001 versus GMG. ns: not significant versus GMG. GM: gentamicin; CG: Control Group; GMG: GM treated Group.

The impact of ZLFs on serum uric acid, urea, and creatinine levels in all analyzed groups was assessed, as seen in Figure 3. A significant increase in creatinine (*p* < 0.001), urea (*p* < 0.01), and uric acid (*p* < 0.01) was observed in rats of the GM treated Group (GMG) (80 mg/kg; b.w), in comparison to the CG’s animals. In addition, compared to the GMG, the rats who were given 200 mg/kg of ZLF extract demonstrated a significant reduction in serum creatinine (*p* < 0.01) and a non-significant decrease in urea and uric acid. However, the dose of 400 mg/kg induced a significant decreased in the concentration of serum creatinine (*p* < 0.001), uric acid (*p* < 0.05), and urea (*p* < 0.01).

#### 2.2.4. The Impact of ZLFs on Urine Creatinine, Uric Acid, and Urea Levels

**Figure 4:**
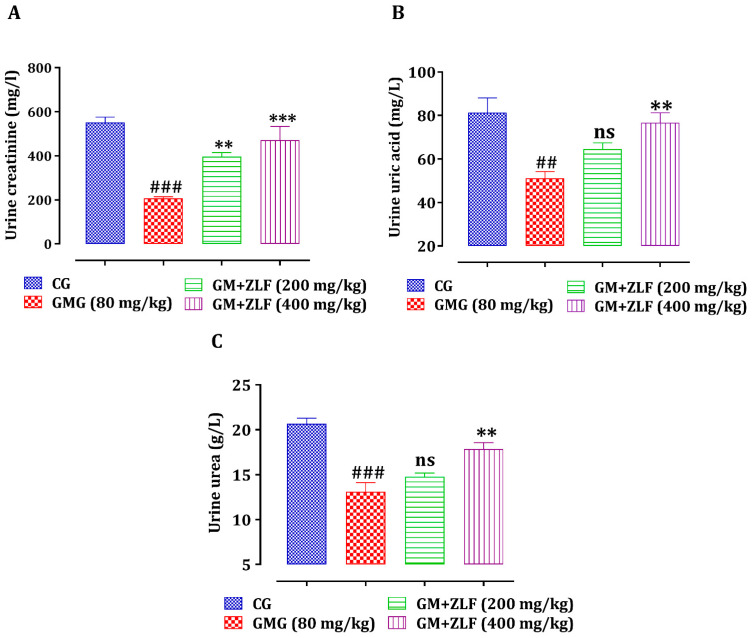
Effect of ZLF’s extract on urine creatinine (**A**), urea (**B**), and uric acid (**C**) in GM-poisoned rats. The data are presented as mean ± SEM, (*n* = 6). ^###^ *p* ≤ 0.001, ^##^ *p* ≤ 0.01 versus CG. ** *p* < 0.01, *** *p* < 0.001 versus GMG. ns: not significant versus GMG. GM: gentamicin; CG: Control Group; GMG: GM treated Group.

The effect of ZLF’s on the urinary concentration of creatinine, uric acid, and urea in GM-intoxicated rats is shown in Figure 4. The urinary concentrations of uric acid, urea, and creatinine decreased significantly (*p* < 0.01, *p* < 0.001, *p* < 0.001, respectively) in GM-treated rats, compared to control rats. The daily intake of ZLF has significantly reversed the nephrotoxic effects of GM, by lowering creatinine, urea, and uric acid levels in the urine.

#### 2.2.5. The Effect of ZLF’s on Creatinine Clearance

**Figure 5:**
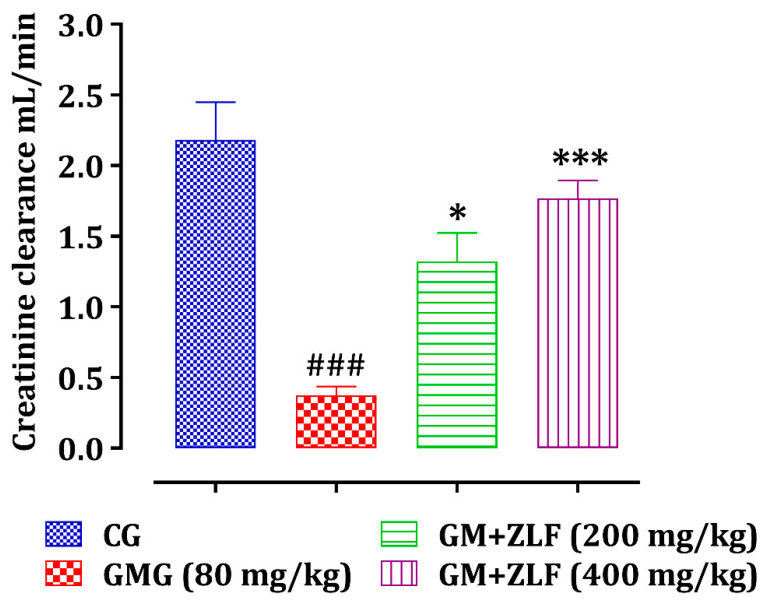
Effect of ZLF’s on creatinine clearance in GM-exposed rats. The data are presented as mean ± SEM, (*n* = 6). ^###^ *p* ≤ 0.001, versus CG. * *p* < 0.05, *** *p* < 0.001 versus GMG. GM: gentamicin; CG: Control Group; GMG: GM treated Group.

As displayed in Figure 5, the effect of ZLF’s on glomerular filtration was evaluated by creatinine clearance calculation in all animals of the study. Injecting GM (80 mg/kg; b.w.) into rats resulted in a substantial (*p* < 0.001) reduction in creatinine clearance. In GMG rats, administration of ZLF’s extract at doses of 200 and 400 mg/kg for 14 days improved creatinine clearance substantially (*p* < 0.05, *p* < 0.001, respectively).

#### 2.2.6. Effect of ZLF’s on Levels of Serum and Urine Albumin

**Figure 6:**
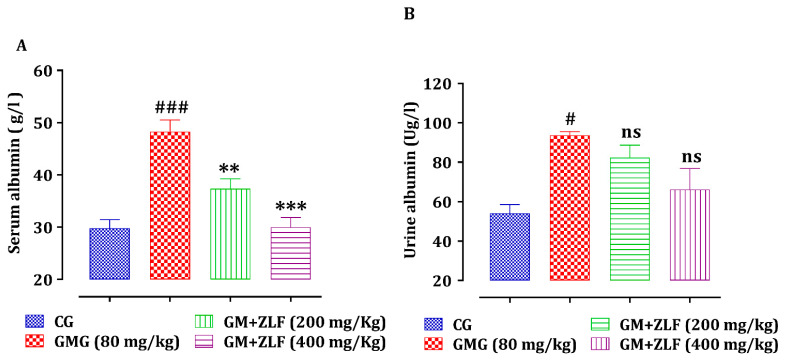
Effect of ZLF’s on serum and urine albumin in GM-exposed rats. The data are presented as mean ± SEM, (*n* = 6). ^#^ *p* ≤ 0.05 ^###^ *p* ≤ 0.001 versus NCG. ** *p* < 0.01, *** *p* < 0.001 versus GMG. ns: not significant versus GMG. GM: gentamicin; CG: Control Group; GMG: GM treated Group.

Figure 6 shows the impact of ZLFs on urinary and serum albumin concentrations in the studied groups. The intraperitoneal injection of GM during 14 days induced a significant increase in serum and urine albumin, respectively, compared to CG. However, the treatment of GM-exposed rats with ZLF has resulted in a significant decrease in serum albumin, and a non-significant decrease in urinary albumin, referring to GMG (80 mg/kg; b.w).

#### 2.2.7. Effect of ZLF’s on ALP and Gamma-GT

**Figure 7:**
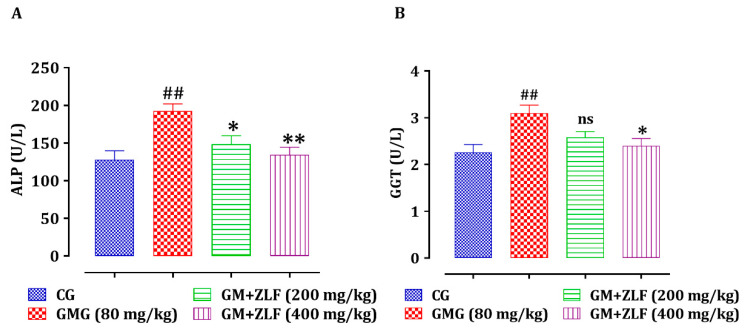
Effect of ZLF’s on serum alkaline phosphatase (A) and gamma-GT (B) in GM-poisoned rats. The data are displayed as mean ± SEM, (*n* = 6). ^##^ *p* < 0.01 related to CG. * *p* < 0.05, ** *p* < 0.01 related to the GMG. ns: not significant versus GMG. GM: gentamicin; CG: Control Group; GMG: GM treated Group.

In comparison to the CG rats, the rats that were given only GM had a substantial improvement (*p* < 0.01) in serum ALP and Gamma-GT (Figure 7), whereas animals of GM + ZLF (400 mg/kg) showed a significant decrease in serum levels of ALP (*p* < 0.01) and Gamma-GT (*p* < 0.05), compared to the GMG (80 mg/kg; b.w). Moreover, animals of the GM + ZLF (200 mg/kg) showed a significant decrease (*p* < 0.05) in serum ALP, but not significant for Gamma-GT compared to animals of GMG (80 mg/kg; b.w).

#### 2.2.8. Effect of ZLF’s on the Kidney Malondialdehydes (MDA) Level

**Figure 8:**
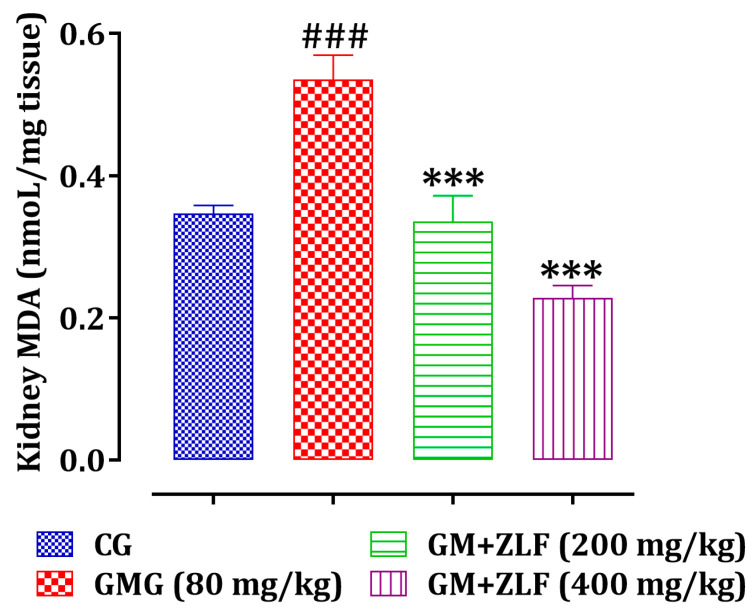
Effect of ZLF’s on kidney MDA level in GM-intoxicated rats. The data are displayed as mean ± SEM, (*n* = 6). ^###^ *p*< 0.01 related to CG. *** *p* < 0.001 versus GMG. GM: gentamicin; CG: Control Group; GMG: GM treated Group.

As observed in Figure 8, a significant increase (*p* < 0.001) in MDA levels in rats exposed to GM compared to rats of the CG, whereas in the groups treated with the ZLF’s at two doses (200 and 400 mg/kg), a significant decrease (*p* < 0.01 and *p* < 0.001, respectively) in the MDA level was marked, referring to rats of the GMG (80 mg/kg; b.w).

#### 2.2.9. Effect of ZLF’s on Serum Electrolytes

The serum concentrations of sodium, potassium, chloride, and calcium for all treated groups are presented in Table 3. The rats had a substantial increase in sodium (*p* < 0.01) and a non-significant improvement in calcium levels after receiving the GM injection. However, a significant decrease in potassium (*p* < 0.05) and a non-significant decrease in chloride were observed compared to rats in the CG. Nevertheless, the administration of the ZLF extract restored the electrolyte changes induced by the intraperitoneal injection of GM in rats. Furthermore, regular pretreatment of rats with ZLF at the dose of 400 mg/kg before injection of GM resulted in a substantial decrease in sodium (*p* < 0.01), and a significant rise in potassium (*p* < 0.05) and chloride against a decrease in calcium compared to GM injected rats. Furthermore, the dose of 200 mg/kg resulted in a non-significant reversal of the deleterious changes caused by GM on the serum electrolyte levels, compared to the GMG (80 mg/kg; b.w).

#### 2.2.10. Effect of ZLF’s on the Renal Histopathological Changes

**Figure 9:**
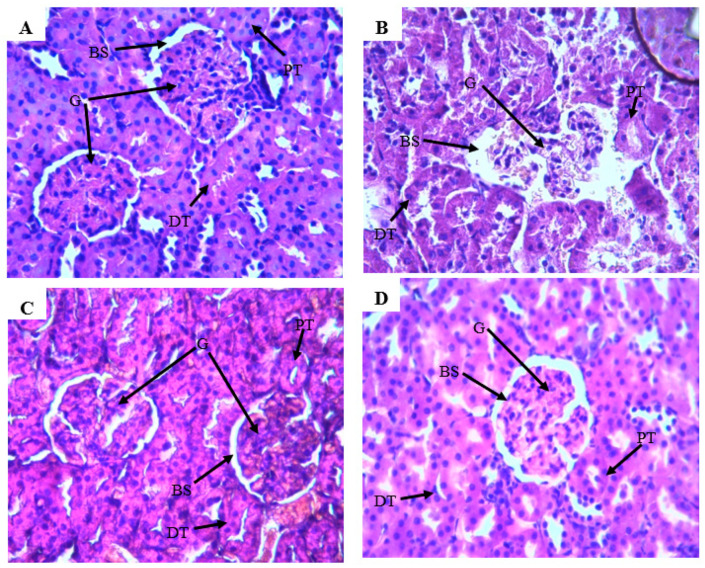
The effect of ZLF extracts on kidney histology in GM-exposed rats. Hematoxylin and eosin staining is used to visualize histological parts, which were then examined under an optical microscope at a magnification of ×40. (**A**) Control group, (**B**) GM treated Group, (**C**,**D**) groups intoxicated with GM and treated with ZLF’s extract at doses 200 and 400 mg/Kg. Glomerulus (G), Distal convoluted tubule (DT), Bowman space (BS), Proximal convoluted tubule (PCT).

The hematoxylin and eosin staining showed that the kidney of the CG has normal renal tubules and glomeruli (Figure 9A). The rats in the GM-intoxicated group showed reduced glomeruli cells, loss of tubular cell components, vascular congestion resulting in epithelial cell atrophy (Figure 9B). In addition, the toxic group’s rats had a deformation of the Bowman space, as well as distortions in the epithelial membrane of the Bowman’s capsule, when opposed to the healthy rats (Figure 9A), which presented a normal histoarchitecture kidney. However, in animals treated with the ZLF extract and injected with GM, there is an improvement in the histoarchitecture of the kidneys compared to the toxic group (Figure 9C,D). Moreover, this improvement in histoarchitecture is comparable to that of the CG (Figure 9A).

## 3. Discussion

In the current research, we evaluated the protective effect of ZLFs against nephrotoxicity caused by intraperitoneal injection of GM in *Wistar* rats. Clinically, GM is a frequently used antibiotic aminoglycoside bactericide to treat severe acute infections. However, owing to the extreme toxic effects on the kidneys, its medicinal application is restricted [27]. Despite its nephrotoxic effects, this aminoglycoside remains the only effective therapeutic alternative against some multi-resistant bacteria [28]. The mechanism of GM nephrotoxicity remains not completely known until now. Nonetheless, both in vitro and in vivo experiments revealed that GM increased reactive oxygen species production [29]. Increased production of free radicals can degrade some structural macromolecules, causing cell damage induction and tubular necrosis by multiple mechanisms, including lipid peroxidation of cell membranes, DNA damage, and protein denaturation [29,30]. In the results of this study, the daily GM intake has induced a decrease in body weight gain, and an increase in relative kidney weight, urinary volume, and water intake. This can be attributed to the accumulation of GM in the renal tubules, resulting in swelling of the kidneys and kidney damage [31]. Accumulation of GM in kidney tissue results in damage of tubular cells resulting in dehydration and thus increased water intake and urinary volume and decrease in the body weight gain [31]. In addition, the GM has provoked a substantial rise in serum urea, uric acid, and creatinine, as well as their decline in urine, and this biochemical disorder is a witness to severe functional impairment of the kidneys [32]. During renal dysfunction, the kidney’s clearance towards creatinine (a no protein waste of creatinine phosphate metabolism) is reduced due to the reduction of glomerular filtration. In addition, a high level of urea results in kidney dysfunction [33]. Increases in serum sodium and calcium levels and decreases in chloride and potassium were also observed in GM-treated rats. This might be attributed to the fact that GM affects the membrane, which carries the brush border of epithelial cells and basolateral membranes, leading to electrolyte imbalance [31]. Besides, lipid peroxidation in the kidneys tissue has been mentioned in several studies as the destructive process of kidney function due to the injection of GM [30]. Injection of GM to rats during 14 days of treatment causes abnormal changes in kidney tissue such as a reduced cell in the glomeruli, loss of cellular tubular constituents, vascular congestion causing atrophy of epithelial cells, distortions of the epithelial membrane of the Bowman capsule, and deformation of Bowman space. In addition to the nephrotoxicity, the results show that GM can also induce hepatotoxicity. Moreover, an increase in GGT, ALP, and albumin (albumin is primarily synthesized in the liver) serum levels are the biomarkers of hepatotoxicity [34]. The GGT activity is localized in the hepatocytes membranes, and its increase in the blood is often caused by leakage of hepatocytes. Injecting GM also causes hepatotoxicity, which contributes to damage of the hepatocyte membranes, and then an increase in the blood GGT. These abnormalities in biochemical parameters and tissue damage produced by the GM are consistent with previously published work [12,35,36,37]. However, the daily administration of the ZLF aqueous extract 3 h before the injection of GM significantly restored these disorders provoked by the GM. The effect of the plant extract has been dose-dependent with the best effect observed with the dose of 400 mg/kg.

Several studies have shown that reducing oxidative stress is one of the possible mechanisms to protect the kidneys against the oxidative stress produced by the GM [31,38,39,40]. It has been shown that polyphenols, flavonoids reduce the nephrotoxicity of GM via the increase in the antioxidant enzymatic activity, decrease in the lipid peroxidation, scavenge the free radicals, and improve tissue architecture of the kidney [41,42]. Indeed, our finding shows that the aqueous extract of ZLF is rich in polyphenolic compounds such as ferulic acid, hydroxytyrosol, gallic acid, catechin, vanillic acid, quercetin *p*-coumaric acid, naringenin, rutin, and 3-hydroxycinnamic acid, according to the HPLC-DAD study. These bioactive compounds have been synthesized and used by plants to protect against pathogens agents [43], and there are well known for their antioxidant power [44]. Based on these results, it seems that our extract relying on these bioactive molecules activities protects the kidneys against GM nephrotoxicity by trapping free radicals produced by GM metabolism.

## 4. Materials and Methods

### 4.1. Reagents

GM was purchased from the pharmacy. Trichloroacetic acid (TCA) and Thiobarbituric acid (TBA) were acquired from the Sigma Aldrich Company (St. Louis, MO, USA). Creatinine, Uric Acid, Urea, Alkaline Phosphatase (ALP), Gamma-Glutamyl-Transpeptidase (Gamma-GT), Albumin, and electrolytes kits were procured from Biosystems, Spain. All the products used in this investigation were deemed as high quality.

### 4.2. Animals

In this study, male and female rats were used to assess the nephroprotective effect of ZLF aqueous extract. The reason why both sexes were selected in this study is actually to validate previously published protocols similar to our work that used both male and female rats with no justification for choosing to use two different sexes [45,46] and to gather more information about the influence of gender for future studies that could build on ours. In this context, twenty-four Wistar rats (50% male and 50% female, weighing between 160 and 250 g, aged between 9 and 11 weeks) were being used in this study. All animals were separated into groups and placed in polypropylene cages and unrestricted access to food (rich only in macromolecules needed for the rat’s growth) and water. The animals were kept under controlled conditions (23 °C, 12 h of darkness/12 h of light) for 29 days (15 days of acclimatization of the animals, followed by 14 days of treatment).

### 4.3. Plant Material

The fruits of *Zizyphus lotus* L. (Desf) (ZLF’s) were harvested in the Eastern region of Morocco in September 2019, and identified by botanist Mohammed Fennane from Mohammed V University’s scientific institute. The specimen was collected, prepared, and deposited at the Herbarium of the laboratory under the acronym «HUMPOM».

### 4.4. Preparation of the ZLF’s Aqueous Extract

After processing the ZLF’s into powder, 100 g was mixed with 2 L of boiled distillated water (75 °C) and infused under agitation for 20 min. At 60 °C, the resultant solution was condensed using a rotary evaporator under a vacuum. The crude extract was dried and stored at −20° before usage.

### 4.5. Nephroprotective Study

#### 4.5.1. Nephrotoxicity Induction in Rats and Doses Selection of ZLF Extract

In this study, nephrotoxicity was induced in rats using daily intraperitoneal injections of GM, at a dose of (80 mg/kg; b w) during all days of treatment. It is well known that the dose considered is commonly used to induce nephrotoxicity in experimental animals [12,47]. The doses 200 and 400 mg/kg of the ZLF aqueous extract were used in this study, referring to a previous similar approach [47], in which the aqueous ZLF extract produced no detectable toxicity to laboratory animals [25].

#### 4.5.2. Experimental Design

Following the two-week acclimatization duration, the animals were placed into four equal groups with six in each (♂/♀ = 1). The Control Group (CG) was treated only with 10 mL/kg of purified water; GM treated Group (GMG) was treated with purified water (10 mL/kg) and injected intraperitoneally with GM (80 mg/kg; b.w). The remaining groups were treated as follows: the first group (GM + ZLF (200 mg/kg)) received 200 mg/kg while the second (GM + ZLF (400 mg/kg)) received the dose of 400 mg/kg of ZLF’s extract and then injected with GM (80 mg/kg b.w). In those last two groups, the GM injection was made after 3 h of administration of the plant extract, during 14 days of treatment. Animals were controlled and monitored continuously for 2 h after the injection of GM and then only once per 3 h for 14 days of treatment (the gradual decrease in the mobility of the animals and the change in their behavior were observed from the 4th injection of GM up to the last day of treatment, especially in the group injected only by GM). After the last dose, all rats of the experiment were then kept in solitary metabolic cages for the compilation of urine simple for 24 h. The urine samples collected were centrifuged with a centrifugal force of 704× *g*. On days 0, 7, and 14 of treatment, the weights of the animals were measured, respectively, with a Mettler scale (see supplementary file).

#### 4.5.3. Sample Collection

The animals were anesthetized and sacrificed at the end of the experiment, and their blood was collected and centrifuged with a centrifugal force of 704× *g* at 4 °C to extract the serum. The serum was removed and stored at −20 degrees Celsius for future testing. Furthermore, the kidneys were weighed and kept at −20 °C to quantify the quantity of MalonDialdiAldehyde (MDA) in the kidney homogenate (10% *w*/*v*) in sodium phosphate buffer (pH 7.0).

#### 4.5.4. Biochemical Analysis

Several biochemical parameters were evaluated in serum and urine: calcium following the method of NM-BAPTA [48], urea following the enzymatic method [49], creatinine following the method of Jaffe [50], ALP following the method of IFCC without pyridoxal-5-phosphate [51], albumin following the Bromocresol Green method [52], uric acid by the enzymatic colorimetric method [53], and gamma-GT by Szasz/Persijn method [54], sodium, potassium, and chloride measured by DAM method. All biochemical parameters were measured with the COBAS INTEGRA^®^ 400-Plus analyzer.

#### 4.5.5. Creatinine Clearance

Creatinine clearance was calculated to evaluate the glomerular filtration rate, based on serum and urinary creatinine concentration, using the following formula Equation (1):

(1) $$\mathrm{CCL}\;\left(\frac{\mathrm{mL}}{\min}\right)=\frac{\mathrm{Urine}\;\mathrm{creatinine}\;\left(\frac{\mathrm{mg}}{\mathrm{mL}}\right)*\mathrm{Urine}\;\mathrm{flow}\;\left(\frac{\mathrm{mL}}{\min}\right)}{\mathrm{Serum}\;\mathrm{creatinine}\;\left(\frac{\mathrm{mg}}{\mathrm{mL}}\right)}$$

The urine output was calculated using this formula: urine flow (mL/min) = value of urine volume (24 h)/1440 (60 min × 24 h = 1440).

#### 4.5.6. Relative Kidney Weight (RKW)

The animals fasted 12 h before the end of the experiment, on the 15th day before euthanasia; the body weight (g) was recorded and the kidneys were isolated and weighed (g) (absolute organ weight) against the relative organ weight of rats using the formula below Equation (2):

(2) $$\mathrm{RKW}\;\left(\%\right)=\frac{\mathrm{Absolute}\;\mathrm{kidney}\;\mathrm{weight}\;\left(g\right)}{\mathrm{Body}\;\mathrm{weight}\;\mathrm{of}\;\mathrm{the}\;\mathrm{rat}\;\mathrm{in}\;\mathrm{sacrifice}\;\mathrm{day}\;\left(g\right)}$$

#### 4.5.7. Kidney Lipid Peroxidation

The renal lipid peroxidation was assessed using the experimental procedure reported by Bueg and Aust [55]. The quantity of TBARS generated is measured in this test. After preparing the kidney homogenate, 0.5 mL of it was mixed with 0.5 mL of TCA (30 percent *w*:*v*). This mixture was then centrifuged with a centrifugal force of 959× *g* at 4 °C. Later, 1 mL of supernatant was combined with 1 mL of TBA (0.67 percent *w*:*v*) and implanted in hot water (100 °C for 10 min) before being buried in ice. The opacity of the combination was measured using a spectrophotometer calibrated to 535 nm.

The finding was revealed in moles (MDA quantity)/g (tissue), using the following molar extinction coefficient: 1.56 × 10^5^ M^−1^ cm^−1^.

#### 4.5.8. Histopathological Examinations

The kidneys of all animals in the experiment were prepared for histopathological evaluation. The tissues were settled in 10% buffered formalin, embedded in paraffin wax, cut into 3–4 µm chunks, and colored with eosin and hematoxylin. The sections of the kidney histology were then examined under optical microscopy, and the histological photos were taken by camera microscope with × 40 magnification.

### 4.6. HPLC-DAD Analysis

Ferulic acid, quercetin, gallic acid, catechin, vanillic acid, hydroxytyrosol, *p*- coumaric acid, naringenin, rutin, and 3-hydroxycinnamic acid (Sigma-Aldrich, Steinheim, Germany) were used as standards for HPLC-DAD (Agilent Technologies 1260 infinity II) analysis.

HPLC-DAD connected to a UV detector and equipped with a quaternary pump was used to analyze the aqueous extract of ZLF’s, based on the protocol described by [56]. Two solvents were used as a mobile phase, one is 0.1% acidified water and the other is acetonitrile. For separation, we used an Eclipse C18 Zorbax plus C18 column (5 µm, 4.6 × 150 mm) with a column furnace temperature set at 35 °C. The flow rates have been set at 1 mL/min and sample injection volume at 10 µL (100 µg/mL sample concentration). The concentration was calculated based on the spectral match of each compound and its retention time (RT), using the following formula Equation (3):

(3) $$\mathrm{Concentration}\;\left(\frac{µg}{\mathrm{mL}}\right)=\frac{\mathrm{Area}\;\left(\mathrm{sample}\right)}{\mathrm{Area}\;\left(\mathrm{standart}\right)}*100$$

### 4.7. Statistical Analysis

The results were indicated as means ± SEM. The graphical representation and the statistical analysis were executed by Graph Pad Prism 5, San Diego, CA, USA, using ANOVA statistics followed by Tukey’s post hoc test for various comparisons. The difference was contemplated significant if *p* < 0.05.

## 5. Conclusions

Based on the biochemical and histological results, we conclude that the ZLF’s extract has improved the altered parameters during the nephrotoxicity induced by GM. These results provide preclinical experimental arguments, suggesting possible renal protection, which supports the popular use of this plant for kidney problems. Further study of these promising protective effects of ZLF’s extract against GM-Induced acute kidney injury may have a considerable impact on developing clinically feasible strategies to treat patients with renal failure.

## Figures and Tables

**Table 1:** HPLC–DAD data of the polyphenolic compounds detected in ZLF aqueous extract.

Molecules	RT (Retention Time) (min)	Concentration (µg/mL)
3-hydroxycinnamic acid	9.915	2.27
Catechin	14.959	2.97
Ferulic acid	21.658	137
Gallic acid	4.518	3.7
Hydroxytyrosol	11.939	2.21
Naringenin	23.375	2.76
P-coumaric Acid	16.827	3.07
Quercetin	5.977	8.55
Rutin	18.214	2.32
Vanillic acid	22.785	5.24

**Table 2:** Effect of ZLF’s on growth parameters in rats exposed to GM.

Groups	Weight Gain (g)	Relative Kidney to Body Weight (g)
CG	15.90 ± 3.71	0.31 ± 0.02
GMG (80 mg/kg)	8.40 ± 1.95 ^##^	0.45 ± 0.074 ^###^
GM + ZLF (200 mg/kg)	10.20 ± 1.79 ^ns^	0.37 ± 0.012 **
GM + ZLF (400 mg/kg)	15.40 ± 2.30 **	0.35 ± 0.012 ***

The data is provided as mean ± SEM, (*n* = 6). ^###^ *p* ≤ 0.001, ^##^ *p* ≤ 0.01 related to CG. *** *p* < 0.001, ** *p* < 0.01 compared to the GMG. ^ns^: not significant compared to the GMG. GM: gentamicin; CG: Control Group; GMG: GM treated Group.

**Table 3:** Effect of ZLF’s extract on serum sodium, potassium, chloride, and calcium levels in rats exposed to GM.

Groups	Sodium (mmol/L)	Potassium (mmol/L)	Chloride (mmol/L)	Calcium (mg/L)
CG	134.00 ± 2.38	5.33 ± 0.80	105.00 ± 1.73	89.75 ± 11.97
GMG (80 mg/Kg)	142.75 ± 1.71 ^##^	3.20 ± 0.14 ^#^	103.00 ± 2.64 ^ns^	95.70 ± 4.61 ^ns^
GM + ZLF (200 mg/Kg)	138.75 ± 2.62 ^ns^	3.90 ± 0.28 ^ns^	103.00 ± 2.08 ^ns^	93.53 ± 0.68 ^ns^
GM + ZLF (400 mg/Kg)	134.5 ± 1.75 **	4.80 ± 0.52 *	104.00 ± 1.15 ^ns^	90.61 ± 7.77 ^ns^

Values are Mean ± SEM, *n* = 6. ^##^ *p*< 0.01, ^#^ *p*< 0.01 versus CG. * *p* < 0.05, ** *p* < 0.01 versus GMG. ^ns^: not significant versus GMG or CG. GM: gentamicin; CG: Control Group; GMG: GM treated group.

## Data Availability

Data are available upon reasonable request.

## Supplementary Materials

The following are available online at, Figure S1: Bodyweight developpement during the experimental period.

## Sample Availability

Samples of the compounds are available from the authors.
