# Supplementary figures and images for: The Nephroprotective Effect of Zizyphus lotus L. (Desf.) Fruits in a Gentamicin-Induced Acute Kidney Injury Model in Rats: A Biochemical and Histopathological Investigation
Source: Molecules. 2021 Aug 8;26(16):4806. doi: 10.3390/molecules26164806 (PMC8401527; doi:10.3390/molecules26164806)

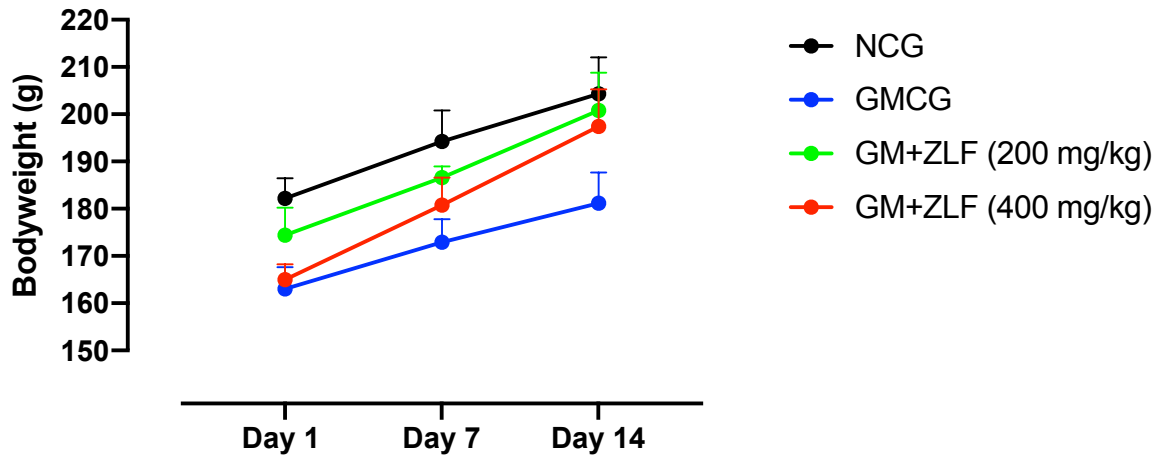

Figure S1. Bodyweight developpement during the experimental period

Supplement: Supplementary file 1 [file molecules-26-04806-s001.zip › molecules-1273976-supplementary.pdf]
